# Brazilian dietary guidelines: interfaces with the mother and child hospital context

**DOI:** 10.1590/S2237-96222024v34e20240391.en

**Published:** 2025-05-02

**Authors:** Simone de Pinho Ferreira Azevedo, Jorginete de Jesus Damião, Inês Rugani Ribeiro de Castro

**Affiliations:** 1 Fundação Oswaldo Cruz, Instituto Fernandes Figueira, Rio de Janeiro, RJ, Brazil; 2 Universidade do Estado do Rio de Janeiro, Departamento de Nutrição Social, Rio de Janeiro, RJ, Brazil

**Keywords:** Diet, Healthy, Tertiary Healthcare, Hospital Care, Nutritional Sciences, Health Promotion, Dieta Saludable, Atención Terciaria de Salud, Atención Hospitalaria, Ciencias de la Nutrición, Promoción de la Salud

## Abstract

**Objective:**

To develop a matrix with potential interfaces between the principles and recommendations of the Brazilian dietary guidelines and food and nutrition care actions developed in the mother and child hospital environment.

**Methods:**

A theoretical-conceptual study that included: reading and extracting excerpts from the Dietary Guidelines for the Brazilian Population and the Dietary Guidelines for Brazilian Children Under 2 Years of Age; mapping, through on-site observation of the care spaces of a maternal and child hospital (inpatient units, outpatient units, human milk bank, lactation room, hospital food and nutrition unit and self-service restaurant/canteen/cafeteria for patient companions); identification and systematization of potential interfaces between food and nutrition care actions in the selected spaces and the principles and recommendations of the Dietary Guidelines; preparation of preliminary versions of the matrix and its assessment by panels of experts; preparation of the final version of the matrix.

**Results:**

The matrix is ​​composed of two sections: one, referring to the Brazilian Population Guidelines and the other, to the Child Guidelines. Different opportunities for materializing the Dietary Guidelines were identified in the matrix, including food and nutrition education in different spaces, creation of menus aligned with the Dietary Guidelines for patients and workers, reorganization of spaces to promote commensality and restriction clauses on ultra-processed foods in food and nutrition units.

**Conclusion:**

The matrix presents necessary interfaces for the implementation of the Dietary Guidelines in the hospital context, highlighting their potential to support the promotion of adequate and healthy eating, as well as to qualify food and nutrition care in this space.

## Resumen

**Objetivo:**

Desarrollar una matriz con posibles interfaces entre los principios y recomendaciones de las Guías Alimentarias Brasileñas y las acciones de atención alimentaria y nutricional desarrolladas en el ambiente hospitalario materno infantil.

**Métodos:**

Estudio teórico-conceptual que incluyó: lectura y extracción de extractos de la Guía Alimentaria para la Población Brasileña y de la Guía Alimentaria para Niños Brasileños Menores de 2 Años; mapeo, a través de la observación in situ de los espacios de atención de un hospital materno infantil (unidades de internación, ambulatorios, banco de leche humana, sala de lactancia, cocina dietética y restaurante/comedor/cafetería de autoservicio para acompañantes); identificación y sistematización de posibles interfaces entre las acciones de cuidado alimentario y nutricional en los espacios seleccionados y los principios y recomendaciones de las Guías; elaboración de versiones preliminares de la matriz y su evaluación por paneles de expertos; preparación de la versión final de la matriz.

**Resultados:**

La matriz está compuesta por dos secciones: una, referida a la Guía para la Población Brasileña y la otra, a la Guía para Niños. En la matriz se identificaron diferentes oportunidades para la materialización de las Guías Alimentarias, entre ellas la educación alimentaria y nutricional en diferentes espacios, la creación de menús alineados a las Guías Alimentarias para pacientes y trabajadores, la reorganización de espacios para promover la comensalidad y cláusulas de restricción para alimentos ultraprocesados ​​en las unidades de alimentación y nutrición.

**Conclusión:**

La matriz presenta interfaces necesarias para la implementación de las Guías Alimentarias en el contexto hospitalario, destacando su potencial para apoyar la promoción de una alimentación adecuada y saludable, así como para calificar la atención alimentaria y nutricional en este espacio.

## Introduction

Adequate and healthy eating is a topic of interest on the public policy agenda of all countries (1). The World Health Organization and the Food and Agriculture Organization of the United Nations advise countries to develop and update food-based dietary guidelines (2). In keeping with international guidelines, Brazil stands out for keeping up to date its *Dietary Guidelines for the Brazilian Population* (3) and *Dietary Guidelines for Brazilian Children Under 2 Years of Age* (4). In addition to serving as instruments for encouraging healthy eating practices at individual and collective levels, these Dietary Guidelines are inductors of public policies that aim to support and protect the health and food and nutrition security of the Brazilian population (5).

These Guidelines are particularly relevant in the hospital environment, since food plays a crucial role in patient recovery and quality of life, directly impacting clinical outcomes, length of stay and costs associated with treatment (6), as well as impacting the health of hospital workers (7,8). In this scenario, promoting adequate and healthy eating, the objective of the Dietary Guidelines, involves both nutritional care of patients and management of the production of meals for patients and workers (8,9).

Despite progress with the implementation of the Brazilian Dietary Guidelines (10-15), a gap persists in the literature. No studies were identified that address promotion of adequate and healthy eating in the hospital context in light of these Dietary Guidelines. To carry out this action, it is essential to consider the specificity of this environment, aiming at articulating clinical and nutritional monitoring. Furthermore, it is essential to reflect on the different food environments present in the hospital context, which may or may not promote adequate and healthy eating for patients’ companions and professionals who work there.

With the aim of contributing to overcoming this gap, in this study a matrix was developed that systematizes potential interfaces between the principles and recommendations of the Dietary Guidelines and food and nutrition care actions identified in the mother and child hospital environment. This context was chosen because, with regard to care for children and adults, it enables comparison of the two current Brazilian Dietary Guidelines with the reality of a hospital environment.

## Methods

### Design

This is a theoretical-conceptual study, which consisted of developing a systematization matrix of potential interfaces between the principles and recommendations of the Dietary Guidelines and food and nutrition care actions in mother and child hospital environments (hereinafter referred to as the matrix).

### Setting

The Fernandes Figueira National Institute for the Health of Women, Children and Adolescents, located in the city of Rio de Janeiro, was chosen as the study setting for mapping care spaces in a mother and child hospital environment (2nd stage of the study), as it is a hospital institution that provides highly complex care and presents different care scenarios, offering varied opportunities for comparison between the two Dietary Guidelines and hospital reality.

### Theoretical and conceptual bases

The *Dietary Guidelines for the Brazilian Population* (3) and the *Dietary Guidelines for Brazilian Children Under 2 Years of Age* (4) were used as theoretical references. These documents are recognized as guiding public policies aimed at promoting adequate and healthy eating. The basis used for the structure of the matrix was the model that analyzed interfaces between the *Dietary Guidelines for the Brazilian Population* and the practice of nutritionists in the area of ​​collective feeding (16). The choice of that model is justified by the absence of similar models intended for the hospital context.

### Stages of the study

The first step in building the matrix consisted of reading the Dietary Guidelines. After exploratory readings, the principles and recommendations of each document were analyzed, from which excerpts containing relevant information for promoting adequate and healthy eating were extracted. Key words/key ideas of potential interfaces between the principles and recommendations of the Dietary Guidelines and food and nutrition care actions in the hospital environment were identified in these excerpts.

The second stage, which comprised the mapping of care spaces, occurred through the observation of these spaces and aimed to identify those that had potential interfaces with food and nutrition care actions, namely: inpatient units (pediatrics, pediatric surgery, expectant mothers, gynecology, rooming-in and intermediate nursery wards), outpatient units (pediatrics, prenatal, gynecology and child care), human milk bank, lactation room, hospital food and nutrition unit (responsible for preparing patients’ meals), and self-service restaurant/canteen/cafeteria, which sersves employees, companions and other costumers. The following were excluded: Intensive Care Units and units for critically ill patients, due to the specificity of the nutritional treatment used in these environments; Pediatric Infectious Diseases ward, due to restricted access; the Interdisciplinary Home Care Program, due to the fact that food and nutrition care is mediated by the home context and dynamics, going beyond the scope of the study; the hospital laboratories, genetics center and operating theaters, based on the understanding that they would not have an interface with food and nutrition care.

The third stage consisted of identifying the potential interfaces between food and nutrition care actions (developed or to be developed) in the selected spaces and the excerpts from the Dietary Guidelines, and also the actors involved in these actions: food and nutrition unit workers; multidisciplinary care team; hospital managers; nutritionists. Then, these interfaces were systematized. This stage was organized into four substages: 

### Preparation of the preliminary version of the matrix

Based on their professional and academic experiences, the authors created the first version of the matrix with potential interfaces between the Dietary Guidelines and care spaces. 

### First specialist panel 

The preliminary version of the matrix was submitted to a panel of experts (17), conducted in remote workshop format whereby the panel was recorded with the consent of the participants. Of the 20 experts invited, 12 researchers and professionals from different Brazilian institutions took part, with theoretical knowledge and/or practical experience on the subject, including six professors from four higher education institutions in the state of Rio de Janeiro, three nutritionists researching the area of ​​nutrition, two nutritionists working in the Institutional Development Support Program of the Brazilian National Health System and a technical advisor from the Ministry of Health. The participants were separated into two groups. Initially, they were asked to describe their general impressions about the matrix, starting from the following triggering question: “Considering potential interfaces between the principles and recommendations of the Brazilian dietary guidelines and food and nutrition care actions in the mother and child hospital environment, what are your general impressions about the matrix?”. They were then invited to contribute with suggestions about content to be included, excluded, edited or detailed. The suggestions made by the first panel were analyzed and incorporated into the matrix, and its second version was then produced. In order to complement the suggestions received in the first panel, a second round of opinions took place in order to evaluate the new version of the matrix.

### Second specialist panel 

The eight experts who had not participated in the first panel were invited to give their opinion on the second version of the matrix asynchronously. Of these, three experts from different universities with different profiles (public health, public policies, experience in preparing the *Dietary Guidelines for Brazilian Children Under 2 Years of Age*) participated in this stage. Their opinions were gathered by their filling out a form containing the following items: general impressions about the matrix and, for each excerpt, suggestions for inclusion, exclusion and editing of content.

### Preparation of the final version of the matrix

The matrix was revised based on the experts’ contributions, incorporating new content and suggestions, so that its final version was prepared.

## Results

The matrix is ​​composed of two sections: the first, referring to the *Dietary Guidelines for the Brazilian Population* and the second, referring to the *Dietary Guidelines for Brazilian Children Under 2 Years of Age*. The structure of each section is presented in Table 1. Each section of the matrix can be viewed in full at http://bit.ly/4hrcih0 e https://bit.ly/4eXSVu4.

**Table 1:** Structure of the sections of the systematization matrix for potential interfaces between the principles and recommendations of the Dietary guidelines and food and nutrition care actions in the mother and child hospital environment

Elements extracted from the *Dietary Guidelines for the Brazilian Population*			Food and nutrition care actions according to care spaces					
Chapter	Excerpt from the text	Key words/key ideas - meaning core (in relation to the entire excerpt or fragment of it in bold text)	Inpatient units	Outpatient units	Hospital food and nutrition unit (responsible for preparing patients’ meals)	Self-service restaurant/canteen (for employees and other customers) and cafeteria for those accompanying hospitalized patients	Lactation room	Human milk bank
Elements extracted from the *Dietary Guidelines for Brazilian Children Under 2 Years of Age*			Food and nutrition care actions according to care spaces					
Chapter	Excerpt from the text	Key words/key ideas - meaning core (in relation to the entire excerpt or fragment of it in bold text)	Inpatient units	Outpatient units	Hospital food and nutrition unit (responsible for preparing patients’ meals)	Self-service restaurant/canteen (for employees and other customers) and cafeteria for those accompanying hospitalized patients	Lactation room	Human milk bank

The following can be highlighted as opportunities for the materialization of the Dietary Guidelines in inpatient units and outpatient units: when preparing dietary prescriptions and dietary plans, consider the patient’s dietary, cultural and social habits and preferences, in addition to their nutritional needs and clinical characteristics, focusing not only on nutrients, but also on foods and food combinations; establish internal dialogue mechanisms and with other points of the Health Care Network to continue care after hospital discharge, with special attention to socially vulnerable families; provide guidance at the time of hospital discharge based on the recommendations of the Dietary Guidelines. 

Regarding the period of inpatient stay, the matrix highlights the importance of providing appropriate space and furniture for eating meals together (e.g. table for eating meals collectively). Particularly, in relation to pediatric inpatient units, it is worth highlighting the importance of doing playful activities in these wards, in order to reduce the time children are exposed to screens. In outpatient units, there are important interfaces with the Dietary Guidelines: actions to encourage breastfeeding and food and nutrition education actions in light of the Dietary Guidelines in waiting rooms, offering support to people who are breastfeeding, guiding them on aspects related to breastfeeding and encouraging them, whenever necessary.

An activity present in the matrix that is common to the two food and nutrition units included in it is to carry out environmental sustainability actions in the meal production process. Examples of this are purchasing organic and agroecological foods from family farming, replacing plastic bags for packaging cutlery with paper bags, and implementing procedures aimed at rational use of water and adequate waste treatment.

In the hospital food and nutrition unit, a central interface with the Dietary Guidelines is the development of balanced menus that include healthy foods and meals. In this area of ​​care, the inclusion, in the terms of reference, of restriction clauses in relation to ultra-processed foods stands out, allowing their use only in specific cases.

In the self-service restaurant/canteen and cafeteria for companions, the alignment of the terms of reference for contracting these services with the Dietary Guidelines includes providing for the obligation for these places to offer healthy options and for there to be no advertising and promotion of ultra-processed foods. The matrix also includes food and nutrition education actions, such as providing service users with recipes that include a variety of unprocessed or minimally processed foods. Another action in these spaces is to offer, upon dietary prescription, food preparations that meet the specific needs of users.

In the lactation room service, an action in line with the Dietary Guidelines is to ensure that diet identification labels do not display the brand names of infant formulas and enteral diets. At the human milk bank, providing educational materials related to postpartum women’s diet and complementary feeding for their babies is a way of materializing the Dietary Guidelines in this space.

## Discussion

The matrix demonstrates that, in addition to being useful for the healthy population, the recommendations of the Dietary Guidelines can be translated into the hospital context and its different spaces.

In inpatient units, as in outpatient units, the interface shown in the matrix between humanized care and the recommendations of the Dietary Guidelines is undeniable. Service users lose their privacy and freedom in the hospital environment and this new and complex experience sometimes translates into distress and discontent (18). In this context, all practices and processes carried out in the hospital environment must aim to achieve humanized care (19,20). 

The National Food and Nutrition Policy indicates that practices involving reception of service users need to consider food and nutrition as determinants of health, taking into account the subjectivity and complexity of eating behavior (21). From this perspective, the matrix highlights listening, by indicating that the patient’s eating, cultural and social habits and preferences must be taken into account in their food prescriptions or dietary plans, which converges with one of the principles of the Dietary Guidelines: food is more than just nutrient intake (3,4).

Still on humanized care, humanization policies in hospitals must view the human dimension of food as an element of identity and sociability (19). The matrix contributes to this reflection by identifying the need for an appropriate place for patients to have meals within inpatient units, as well as provision of adequate furniture, so that they can have their meals outside their beds and in collective spaces. In this aspect, the matrix dialogues with the Dietary Guidelines on the act of eating and commensality.

Coordination of the hospital’s health team with teams from different levels of health care to carry out nutritional monitoring appears as an important action foreseen in the matrix at the time of hospital discharge. In this context, the dimension of comprehensive care thought of in “macro” terms emerges, referred to as “expanded integrality” (22). It is characterized by health network, institutional, intentional and procedural articulation, of multiple “focused integralities” (health center, health team, specialty outpatient clinic or hospital) based on people’s real needs (22). Therefore, the moment of discharge, both for hospitalized patients and those treated in outpatient clinics, must be understood as a privileged moment for ensuring continuity of nutritional care in other services.

From the perspective of comprehensive hospital care, care in health organizations, especially in hospitals, is, by nature, multidisciplinary, that is, it depends on the combination of the work of several professionals with different training (23). The matrix dialogues with this perspective by foreseeing, upon hospital discharge, the participation of the social work sector to support families in situations of food insecurity. This action present in the matrix is ​​aligned with the Dietary Guidelines, which encourage the development of strategies for the promotion and realization of the human right to adequate food (3,4).

Considered as spaces that integrate the hospital food environment, the self-service restaurant/canteen and cafeteria for companions, as well as the hospital food and nutrition unit, appear in the matrix with great potential for the materialization of the recommendations of the Dietary Guidelines. This is because hospitals, in addition to being institutions that play a fundamental role in health recovery, are places where many people work, which makes them strategic for interventions related to the prevention and control of chronic non-communicable diseases and the promotion of adequate and healthy eating, since many of these workers eat most of their meals in this space (7,8,24).

Therefore, it is necessary to take a careful look at the food environment of these institutions, as, in these places, factors can interfere with nutrition and contribute to the increase in the prevalence of overweight, such as lack of access to healthy snacks and meals and exposure to aggressive advertising of ultra-processed foods (25,26). In order to mitigate these factors, actions must be implemented to make these food environments healthier. 

The presence of processed and ultra-processed foods, including those for special purposes, is a reality in hospital food (27,28). In the lactation room, infant formulas and enteral diets are handled, as well as mucilages and chocolate drinks used to prepare baby feeding bottles. It is worth noting that prescribing ultra-processed foods for hospitalized children may be associated with the eating habits that these children adopt in their homes. In Brazil, prevalence of ultra-processed food consumption among children aged 6 to 23 months is 80.5% (29). It is necessary to consider that the period of hospitalization represents a delicate moment for making changes in eating habits, as, in addition to the child being in an unfamiliar environment, there are tensions related to the period of hospital stay (19). However, food and nutrition education based on the recommendations of the Dietary Guidelines, whether carried out with the children themselves or with their companion, should not be neglected, as the immediate consequences of inadequate nutrition at this stage are related to inadequate growth, malnutrition, excess weight, among other harms to the child’s health (30).

It is widely recognized that many patients have specific dietary therapy needs, which makes the provision of adequate nutritional support a priority (27). In this context, use of ultra-processed foods for special purposes, such as infant formulas and enteral diets, is common in specialized pediatric care units. It is essential to implement actions that comply with the Brazilian Norm for the Marketing of Food for Infants and Young Children, teats, pacifiers and feeding bottles (31), which includes not displaying brand names. In line with these actions, the lactation room should not expose patients to the trade names of enteral diets and infant formulas used by them.

Not using ultra-processed foods is also a challenge in the hospital food and nutrition unit. Biscuits, concentrated juices, gelatin and industrialized cakes are frequently present in hospitals (27). Low cost and practicality are highlighted as aspects that lead to the use of these foods (27). Insufficient workforce, limited financial resources and lack of adequate equipment can also contribute to use of ultra-processed foods in hospitals. However, a menu based on the principles of the Dietary Guidelines can become a nutritionist’s ally. Prioritizing unprocessed or minimally processed foods on the menu, limiting the use of processed foods and avoiding ultra-processed foods are possible alternatives that are in line with the Dietary Guidelines.

As shown in the Results section, sustainability actions are emphasized in the matrix in its food and nutrition units. From this perspective, it is crucial to stimulate relationships with local food suppliers and/or representatives of family farming, contributing to strengthening the local economy and sustainable rural development (32).

Regarding obstacles to the adoption of the Dietary Guidelines recommendations included in the matrix, the use of screens stands out, especially by children. The matrix incorporates recommendations regarding restricting the use of screens in childhood (33). Children under the age of 2 should not have access to these resources; between 2 and 5 years old, maximum screen exposure of one hour per day is recommended and between 6 and 10 years old, up to two hours is recommended (33). This recommendation aims not only to reduce children’s exposure to advertisements, but also to avoid distractions during meals (33). In the same direction, the matrix suggests carrying out playful activities in pediatric wards to reduce screen time. However, for this action, the support of professionals and the provision of adequate space are necessary points and can represent barriers to its implementation. In this case, the matrix can be used as an instrument to raise awareness in negotiation processes to change routines and structures in the hospital environment.

Another action that may encounter obstacles in its implementation is the provision of nutritional guidance aimed at postpartum women and complementary feeding of their babies at the human milk bank, as this place, the central objective of which is to support breastfeeding and the clinical management of breastfeeding and newborn nutrition, does not usually have professionals to provide such guidance.

Although nutritionists are protagonists of actions to promote adequate and healthy eating, commitment of all health professionals is essential so that the Dietary Guidelines can be disseminated and implemented. Interdisciplinary teams, by understanding the recommendations of the Dietary Guidelines, can incorporate them into their work, multiplying them, encouraging adoption of appropriate and healthy eating practices by the population served and by workers in the hospital. Therefore, training professionals to disseminate dietary guidelines is essential for the success of their implementation (15). 

When considering a mother and child hospital as the basis for its being built, the matrix refers to the importance of concentrating on this audience, as the quality of food during pregnancy and the first years of life impacts on child development and adult life, since the intrauterine period and the first two years of life are phases that are particularly sensitive to metabolic and nutritional factors, which can cause health consequences in the short and long term (28). In this context, it is necessary to disseminate information and propose actions aimed at breastfeeding and adequate and healthy eating, which reinforces the importance of the matrix.

The matrix presents barriers and challenges that need to be overcome in order for it to become a reality, that is, so that the actions listed in it materialize. The feasibility of its implementation does not only depend on the engagement and commitment of workers, but mainly on the hospital management team, which has the decision-making power to implement these actions.

With the intention of translating the recommendations of the Dietary Guidelines to the hospital setting, the matrix can be considered an important tool for guiding not only the performance of health professionals in nutritional care within the hospital context of the Brazilian National Health System or the private sector, but also to support hospital management in creating mechanisms that effectively implement the guidelines of the Brazilian dietary guidelines.

Some limitations must be considered when interpreting the results. The first lies in the size of the matrix. As it covers excerpts from both Dietary Guidelines, its two sections are extensive. However, the matrix is ​​intended to be a detailed product so that each service can make use of the part that it considers necessary or priority. Another limitation is the absence, in the matrix, of the emergency department and self-service machines. On the other hand, it is worth noting that it can be adapted to each reality, and can be complemented with other services and hospital sectors relevant in each context.

The matrix presents necessary interfaces for the implementation of the Dietary Guidelines in the mother and child hospital context, and in other similar contexts, highlighting their potential for promoting adequate and healthy eating, as well as for qualifying food and nutrition care in this space.

## Data Availability

Not applicable.
