# Supplementary material for: Brazilian dietary guidelines: interfaces with the mother and child hospital context
Source: Epidemiol Serv Saude. 2025 May 2;34:e20240391. doi: 10.1590/S2237-96222024v34e20240391.en (PMC12052323; doi:10.1590/S2237-96222024v34e20240391.en)
Supplement: Translation

# Translation: Guias alimentares brasileiros: interfaces com o contexto hospitalar materno-infantil

**DOI:** 10.1590/S2237-96222024v34e20240391.pt

**Authors:** Simone de Pinho Ferreira Azevedo, Jorginete de Jesus Damião, Inês Rugani Ribeiro de Castro

**Affiliations:** 1 Fundação Oswaldo Cruz, Instituto Fernandes Figueira, Rio de Janeiro, RJ, Brasil; 2 Universidade do Estado do Rio de Janeiro, Departamento de Nutrição Social, Rio de Janeiro, RJ, Brasil

**Keywords:** Alimentação Saudável, Atenção Terciária à Saúde, Assistência Hospitalar, Nutrição, Promoção da Saúde

## Abstract

**Objetivo:**

Desenvolver matriz com potenciais interfaces entre os princípios e as recomendações dos Guias Alimentares brasileiros e as ações de cuidado alimentar e nutricional desenvolvidas no ambiente hospitalar materno-infantil.

**Métodos:**

Estudo de cunho teórico-conceitual que abarcou: leitura e extração de trechos do Guia Alimentar para a População Brasileira e do Guia Alimentar para Crianças Brasileiras Menores de 2 anos; mapeamento, por meio de observação in loco, dos espaços de cuidado de um hospital materno-infantil (unidades de internação, ambulatórios, banco de leite humano, lactário, cozinha dietética e restaurante com autosserviço/cantina/refeitório dos acompanhantes); identificação e sistematização das potenciais interfaces entre as ações de cuidado alimentar e nutricional nos espaços selecionados e os princípios e recomendações dos Guias; elaboração de versões preliminares da matriz e sua apreciação em painéis de especialistas; elaboração da versão final da matriz.

**Resultados:**

A matriz é composta por duas seções: uma, referente ao Guia da População Brasileira e outra, ao Guia da Criança. Diferentes oportunidades para a materialização dos Guias Alimentares foram identificadas na matriz, dentre elas a educação alimentar e nutricional nos diferentes espaços, a elaboração de cardápios alinhados aos Guias para pacientes e trabalhadores, a reorganização de espaços para promover a comensalidade e cláusulas de barreira para alimentos ultraprocessados nas unidades de alimentação e nutrição.

**Conclusão:**

A matriz apresenta interfaces necessárias para a concretização dos Guias Alimentares no contexto hospitalar, evidenciando o seu potencial para apoiar a promoção da alimentação adequada e saudável, bem como para a qualificação do cuidado em alimentação e nutrição neste espaço.

## Introdução

A alimentação adequada e saudável é tema de interesse na agenda de políticas públicas dos países (1). A Organização Mundial da Saúde e a Organização das Nações Unidas para a Alimentação e Agricultura orientam aos países que desenvolvam e atualizem guias alimentares baseados em alimentos (2). Em convergência com as diretrizes internacionais, o Brasil se destaca pela atualização do *Guia Alimentar para a População Brasileira* (3) e do *Guia Alimentar para Crianças Brasileiras Menores de 2 Anos* (4). Além de servirem como instrumentos para incentivar práticas alimentares saudáveis nos âmbitos individual e coletivo, esses Guias são indutores de políticas públicas que visam apoiar e proteger a saúde e a segurança alimentar e nutricional da população brasileira (5).

No ambiente hospitalar, essas diretrizes são particularmente relevantes, uma vez que a alimentação desempenha papel crucial na recuperação e na qualidade de vida dos pacientes, impactando diretamente os desfechos clínicos, a duração da internação e os custos associados ao tratamento (6), e também, na saúde dos trabalhadores que nele atuam (7,8). Nesse cenário, a promoção da alimentação adequada e saudável, objetivo dos Guias, envolve tanto o cuidado nutricional dos pacientes, quanto a gestão da produção de refeições para pacientes e trabalhadores (8,9).

Apesar dos avanços na implementação dos Guias Alimentares brasileiros (10-15), uma lacuna persiste na literatura. Não foram identificados estudos que abordem a promoção da alimentação adequada e saudável no contexto hospitalar à luz desses Guias. Para efetivar essa ação, é essencial considerar a especificidade desse ambiente, visando à articulação entre o acompanhamento clínico e o nutricional. Além disso, é fundamental refletir sobre os diversos ambientes alimentares presentes no contexto hospitalar, que podem ou não promover uma alimentação adequada e saudável para acompanhantes de pacientes e para os profissionais que atuam nesses locais.

Visando contribuir para a superação dessa lacuna, no presente estudo foi desenvolvida uma matriz que sistematiza potenciais interfaces entre princípios e recomendações dos Guias e as ações de cuidado alimentar e nutricional identificadas no ambiente hospitalar materno-infantil. Este contexto foi escolhido porque, ao atender crianças e adultos, permite o exercício de cotejamento dos dois Guias Alimentares brasileiros vigentes com a realidade de um ambiente hospitalar.

## Métodos

### Desenho do estudo 

Estudo de cunho teórico-conceitual, que consistiu na elaboração de uma matriz de sistematização de potenciais interfaces entre os princípios e as recomendações dos Guias Alimentares e as ações de cuidado alimentar e nutricional em ambiente hospitalar materno-infantil (doravante denominada matriz). 

### Contexto

O Instituto Nacional de Saúde da Mulher, da Criança e do Adolescente Fernandes Figueira, na cidade do Rio de Janeiro, foi escolhido como cenário de estudo para a realização do mapeamento dos espaços de cuidado em um ambiente hospitalar materno-infantil (2ª etapa do estudo), por ser uma instituição hospitalar que possui atendimento de alta complexidade e por apresentar diferentes cenários de cuidado, oferecendo variadas oportunidades de cotejamento entre os dois Guias Alimentares e a realidade hospitalar.

### Bases teóricas e conceituais

Foram adotados como referenciais teóricos o *Guia Alimentar para a População Brasileira* (3) e o *Guia Alimentar para Crianças Brasileiras Menores de 2 anos* (4). Esses documentos são reconhecidos como orientadores de políticas públicas voltadas à promoção da alimentação adequada e saudável. Para a estrutura da matriz, utilizou-se como base o modelo que analisou as interfaces entre o *Guia Alimentar para a População Brasileira* e a prática de nutricionistas na área de alimentação coletiva (16). A escolha desse modelo se justifica pela ausência de modelos similares voltados para o contexto hospitalar.

### Etapas do estudo

A primeira etapa para construção da matriz consistiu na leitura dos Guias Alimentares. Depois de realizadas leituras exploratórias, procedeu-se à análise dos princípios e recomendações de cada documento, a partir dos quais foram extraídos os trechos que contêm as informações relevantes para a promoção da alimentação adequada e saudável. Nesses trechos, foram identificadas palavras/ideias-chave das potenciais interfaces entre os princípios e recomendações dos Guias e as ações de cuidado alimentar e nutricional em âmbito hospitalar.

A segunda etapa, que compreendeu o mapeamento dos espaços de cuidado, ocorreu por meio da observação desses espaços e objetivou identificar aqueles que possuíam potenciais interfaces com ações de cuidado alimentar e nutricional, a saber: unidades de internação (enfermaria de pediatria, de cirurgia pediátrica, de gestantes, de ginecologia, de alojamento conjunto e de berçário intermediário), ambulatórios (de pediatria, pré-natal, ginecologia e de acompanhamento infantil), banco de leite humano, lactário e as seguintes unidades de alimentação e nutrição: cozinha dietética (responsável pelo preparo das refeições dos pacientes) e restaurante com autosserviço/cantina/refeitório, que atende funcionários, acompanhantes e outros clientes. Foram excluídos: unidades de terapia intensiva e de pacientes graves, pela especificidade do tratamento nutricional empregado nesses ambientes; enfermaria de Doenças Infecciosas Pediátricas, pela restrição de acesso; o Programa de Assistência Domiciliar Interdisciplinar, pelo fato de o cuidado alimentar e nutricional ser mediado pelo contexto e pela dinâmica domiciliar, extrapolando o escopo do estudo; os laboratórios, o centro de genética e os centros cirúrgicos, pelo entendimento de que não teriam interface com o cuidado em alimentação e nutrição.

A terceira etapa consistiu na identificação das potenciais interfaces entre as ações de cuidado alimentar e nutricional (desenvolvidas ou por desenvolver) nos espaços selecionados e os trechos dos Guias, e, também, dos atores envolvidos nessas ações: trabalhadores da unidade de alimentação e nutrição; equipe multiprofissional; gestores; nutricionistas. Em seguida, foi realizada a sistematização dessas interfaces. Essa etapa foi organizada em quatro subetapas: 

### Elaboração da versão preliminar da matriz

Com base em suas experiências profissionais e acadêmicas, as autoras elaboraram a primeira versão da matriz com as potenciais interfaces entre os Guias e os espaços de cuidado. 

### Realização do primeiro painel de especialistas

A versão preliminar da matriz foi submetida a um painel de especialistas (17), realizado no formato de oficina remota com a gravação da atividade com o consentimento dos participantes. Dos 20 especialistas convidados, participaram 12 pesquisadores e profissionais de diferentes instituições do país que apresentavam domínio teórico e/ou experiência prática no assunto, sendo seis docentes de quatro instituições de ensino superior do estado do Rio de Janeiro, três nutricionistas pesquisadoras da área da nutrição, duas nutricionistas atuantes no Programa de Apoio ao Desenvolvimento Institucional do Sistema Único de Saúde e uma assessora técnica do Ministério da Saúde. Os participantes foram separados em dois grupos. Inicialmente, foi solicitado que descrevessem suas impressões gerais sobre a matriz, partindo da seguinte pergunta disparadora: “Considerando as potenciais interfaces entre os princípios e as recomendações dos Guias Alimentares brasileiros e as ações de cuidado alimentar e nutricional no ambiente hospitalar materno-infantil, quais as impressões gerais sobre a matriz?”. Na sequência, eles foram convidados a contribuir com sugestões sobre conteúdos a serem incluídos, excluídos, editados ou detalhados. As sugestões do primeiro painel foram analisadas e incorporadas à matriz, sendo, então, produzida sua segunda versão. Com a finalidade de complementar as sugestões recebidas no primeiro painel, uma segunda rodada de opiniões foi realizada para a apreciação da nova versão da matriz.

### Realização do segundo painel de especialistas

Os oito especialistas que não haviam participado do primeiro painel foram convidados a opinar sobre a segunda versão da matriz de modo assíncrono. Desses, três especialistas de diferentes universidades com perfis diversos (saúde coletiva, políticas públicas, experiência na elaboração do *Guia Alimentar para Crianças Brasileiras Menores de 2 anos*) participaram desta etapa. O levantamento de opiniões foi realizado por meio de preenchimento de um formulário contendo os seguintes itens: impressões gerais sobre a matriz e, para cada trecho, sugestões de inclusão, exclusão e edição de conteúdo.

### Elaboração da versão final da matriz

Com base nas contribuições dos especialistas, a matriz foi revisada, incorporando-se novos conteúdos e sugestões, sendo elaborada sua versão final.

## Resultados

A matriz é composta por duas seções: a primeira, referente ao *Guia Alimentar para a População Brasileira* e a segunda, referente ao *Guia Alimentar para Crianças Brasileiras Menores de 2 anos*. A estrutura de cada uma das seções está apresentada na Tabela 1. A íntegra de cada seção da matriz pode ser visualizada em http://bit.ly/4hrcih0 e https://bit.ly/4eXSVu4.

**Tabela 1:** Estrutura das seções da matriz de sistematização de potenciais interfaces entre os princípios e recomendações dos Guias e as ações de cuidado alimentar e nutricional em ambiente hospitalar materno-infantil

Elementos extraídos do *Guia Alimentar da População Brasileira*			Ações de cuidado alimentar e nutricional por espaços de cuidado					
Capítulo	Trecho do texto	Palavras/ideias-chave - núcleo de sentido (referentes a todo o trecho ou ao fragmento em negrito)	Unidades de internação	Ambulatórios	Cozinha dietética (responsável pelo preparo das refeições dos pacientes)	Restaurante com autosserviço/cantina (para funcionários e outros clientes) e refeitório dos acompanhantes de pacientes internados	Lactário	Banco de leite humano
Elementos extraídos do *Guia Alimentar para Crianças Brasileiras Menores de 2 Anos*			Ações de cuidado alimentar e nutricional por espaços de cuidado					
Capítulo	Trecho do texto	Palavras/ideias-chave - núcleo de sentido (referentes a todo o trecho ou ao fragmento em negrito)	Unidades de internação	Ambulatórios	Cozinha dietética (responsável pelo preparo das refeições dos pacientes)	Restaurante com autosserviço/cantina (para funcionários e outros clientes) e refeitório dos acompanhantes de pacientes internados	Lactário	Banco de leite humano

Nas unidades de internação e nos ambulatórios, podem-se destacar como oportunidades para a materialização dos Guias: na prescrição dietética e no plano dietético, considerar os hábitos e as preferências alimentares, culturais e sociais do paciente, além das suas necessidades nutricionais e características clínicas, com foco não somente nos nutrientes, mas também nos alimentos e nas combinações de alimentos; estabelecer mecanismos de interlocução interna e com outros pontos da Rede de Atenção à Saúde para dar continuidade ao cuidado após a alta hospitalar, com atenção especial às famílias vulnerabilizadas socialmente; realizar orientações no momento da alta hospitalar baseadas nas recomendações dos Guias. 

Sobre o período da internação, a matriz evidencia a importância da disponibilização de local e mobiliário apropriados para a realização das refeições em companhia (ex: mesa para consumo coletivo). Particularmente, em relação às unidades pediátricas de internação, merece destaque a realização de atividades lúdicas nessas enfermarias, de forma a reduzir o tempo de exposição das crianças a telas. Nos ambulatórios, são interfaces importantes com os Guias: ações de estímulo ao aleitamento materno e ações de educação alimentar e nutricional à luz dos Guias nas salas de espera, oferecendo apoio à pessoa que amamenta, orientando-a sobre aspectos ligados à amamentação e encorajando-a, sempre que necessário.

Uma atividade presente na matriz que é comum às duas unidades de alimentação e nutrição nela incluídas é a de realizar ações de sustentabilidade ambiental no processo produtivo das refeições. Exemplos disso são a aquisição de alimentos orgânicos e agroecológicos oriundos da agricultura familiar, a substituição de sacos de plástico para embalar talheres por sacos de papel, e a implementação de procedimentos voltados ao uso racional da água e ao tratamento adequado dos resíduos.

Na cozinha dietética, uma interface central com os Guias é a elaboração de cardápios equilibrados que contemplem alimentos e refeições saudáveis. Neste espaço de cuidado, destaca-se a inclusão, no termo de referência, de cláusulas de barreira em relação aos alimentos ultraprocessados, permitindo o uso apenas em casos específicos.

No restaurante com autosserviço/cantina e refeitório dos acompanhantes, o alinhamento do termo de referência para a contratação desses serviços aos Guias Alimentares inclui prever a obrigatoriedade de esses locais oferecerem opções saudáveis e de não haver publicidade e promoção de alimentos ultraprocessados. Na matriz também estão presentes ações de educação alimentar e nutricional, como fornecer aos usuários receitas que contemplem alimentos in natura ou minimamente processados variados. Outra ação nesses espaços é oferecer, mediante prescrição dietética, preparações que atendam necessidades específicas dos usuários.

No serviço de lactário, uma ação alinhada aos Guias é garantir que as etiquetas de identificação das dietas não apresentem os nomes comerciais das fórmulas infantis e de dietas enterais. No banco de leite humano, o fornecimento de materiais educativos relacionados à alimentação da puérpera e à alimentação complementar da criança é uma forma de materializar os Guias nesse espaço.

## Discussão

A matriz demonstra que, além de úteis para a população saudável, as recomendações dos Guias podem ser traduzidas para o contexto hospitalar em seus diferentes espaços.

Nas unidades de internação, como nos ambulatórios, é incontestável a interface mostrada na matriz entre o cuidado humanizado e as recomendações dos Guias. Os usuários perdem sua privacidade e liberdade no ambiente hospitalar e essa vivência nova e complexa por vezes se traduz em aflições e descontentamentos (18). Nesse contexto, todas as práticas e processos realizados no ambiente hospitalar devem visar ao cuidado humanizado (19,20). 

A Política Nacional de Alimentação e Nutrição indica que as práticas de acolhimento precisam considerar a alimentação e nutrição como determinantes de saúde, levando em conta a subjetividade e a complexidade do comportamento alimentar (21). Nessa perspectiva, a matriz evidencia a escuta, ao indicar que, na prescrição alimentar ou no plano dietético, os hábitos e preferências alimentares, culturais e sociais do paciente devem ser levados em consideração, o que converge com um dos princípios dos Guias: a alimentação é mais que ingestão de nutrientes (3,4).

Ainda sobre o cuidado humanizado, as políticas de humanização nos hospitais devem compreender a dimensão humana da alimentação como elemento de identidade e de sociabilidade (19). A matriz contribui para essa reflexão ao identificar a necessidade de um local apropriado para a realização da refeição dos pacientes dentro das unidades de internação, assim como a disponibilização de mobiliário adequado, de forma que eles possam realizar suas refeições fora do leito e em espaços coletivos. Nesse aspecto, a matriz dialoga com as orientações dos Guias sobre o ato de comer e a comensalidade.

A articulação da equipe de saúde do hospital com equipes dos diferentes níveis de atenção à saúde para realizar o acompanhamento nutricional aparece como uma importante ação prevista na matriz no momento da alta hospitalar. Nesse contexto, emerge a dimensão da integralidade da atenção pensada no “macro”, denominada de “integralidade ampliada” (22). Ela se caracteriza pela articulação em rede, institucional, intencional, processual, das múltiplas “integralidades focalizadas” (centro de saúde, equipe de saúde, ambulatório de especialidades ou hospital) baseando-se nas necessidades reais das pessoas (22). Assim, o momento da alta, tanto para pacientes internados quanto para aqueles atendidos no ambulatório, deve ser compreendido como privilegiado para assegurar a continuidade do cuidado nutricional em outros serviços.

Na perspectiva da integralidade da atenção hospitalar, o cuidado nas organizações de saúde, especialmente nos hospitais, é, por natureza, multidisciplinar, ou seja, depende da conjugação do trabalho de vários profissionais com diferentes formações (23). A matriz dialoga com essa perspectiva ao prever, na alta hospitalar, a participação do setor de assistência social para apoiar famílias em situação de insegurança alimentar. Essa ação presente na matriz está alinhada aos Guias, que estimulam o desenvolvimento de estratégias para a promoção e a realização do direito humano à alimentação adequada (3,4).

Considerados espaços que integram o ambiente alimentar hospitalar, o restaurante com autosserviço/cantina e refeitório dos acompanhantes, assim como a cozinha dietética, aparecem na matriz com grande potencial para a materialização das recomendações dos Guias. Isso porque os hospitais, além de serem instituições que desempenham papel fundamental na recuperação da saúde, são locais de trabalho de muitas pessoas, o que os tornam estratégicos para intervenções relacionadas à prevenção e ao controle das doenças crônicas não transmissíveis e à promoção da alimentação adequada e saudável, uma vez que muitos desses trabalhadores realizam a maior parte das suas refeições nesse espaço (7,8,24).

Assim, torna-se necessário um olhar cuidadoso para o ambiente alimentar dessas instituições, pois, nesses locais, fatores podem interferir na alimentação e contribuir para o aumento da prevalência de excesso de peso, como a falta de acesso a lanches e refeições saudáveis e a exposição à publicidade agressiva de alimentos ultraprocessados (25,26). Para mitigar esses fatores, devem ser implementadas ações que tornem esses ambientes alimentares mais saudáveis. 

A presença de alimentos processados e ultraprocessados, incluindo aqueles para fins especiais, é uma realidade na alimentação hospitalar (27,28). No lactário, ocorre a manipulação de fórmulas infantis e dietas enterais, assim como de mucilagens e de achocolatados utilizados no preparo de mamadeiras. Cabe destacar que a prescrição de alimentos ultraprocessados às crianças internadas pode estar associada aos hábitos alimentares que essas crianças adotam em seus lares. No Brasil, a prevalência de consumo de alimentos ultraprocessados entre crianças de 6 a 23 meses é de 80,5% (29). É preciso considerar que o período de internação representa um momento delicado para realizar alterações no hábito alimentar, pois, além de a criança se encontrar em um ambiente desconhecido, existem as tensões relativas ao período de internação hospitalar (19). Contudo, a educação alimentar e nutricional embasada nas recomendações dos Guias, seja ela realizada com a própria criança ou com seu acompanhante, não deve ser negligenciada, pois as consequências imediatas da alimentação inadequada nessa fase relacionam-se a crescimento inadequado, desnutrição, excesso de peso, dentre outros prejuízos à saúde da criança (30).

É amplamente reconhecido que muitos pacientes apresentam necessidades dietoterápicas específicas, o que torna prioritário o fornecimento de suporte nutricional adequado (27). Nesse contexto, a utilização de alimentos ultraprocessados para fins especiais, como fórmulas infantis e dietas enterais, é frequente em unidades pediátricas de atendimento especializado. É imprescindível a implementação de ações que estejam em conformidade com a *Norma Brasileira de Comercialização de Alimentos para Lactentes e Crianças de Primeira Infância*, bicos, chupetas e mamadeiras (31), o que inclui a não exposição dos nomes comerciais. Em consonância com essas ações, o lactário não deve expor aos pacientes os nomes comerciais das dietas enterais e fórmulas infantis utilizadas por eles.

Na cozinha dietética, a não utilização de alimentos ultraprocessados é também um desafio. Bolachas, sucos concentrados, gelatina e bolos industrializados estão frequentemente presentes nos hospitais (27). O baixo custo e a praticidade são apontados como aspectos que levam ao uso desses alimentos (27). Força de trabalho insuficiente, limitação de recursos financeiros e falta de equipamentos adequados também podem contribuir para o uso dos ultraprocessados nos hospitais. Entretanto, o cardápio baseado nos princípios dos Guias pode se tornar um aliado do nutricionista. Priorizar no cardápio alimentos in natura ou minimamente processados, limitar o uso de alimentos processados e evitar os alimentos ultraprocessados são alternativas possíveis e que vão ao encontro dos Guias. 

Conforme evidenciado na seção de resultados, ações de sustentabilidade são enfatizadas na matriz nas unidades de alimentação e nutrição. Nessa perspectiva, é crucial estimular a relação com fornecedores de alimentos locais e/ou representantes da agricultura familiar, contribuindo para o fortalecimento da economia local e para o desenvolvimento rural sustentável (32).

No tocante aos obstáculos à adoção das recomendações dos Guias contempladas na matriz, o uso de telas se sobressai, principalmente pelas crianças. A matriz incorpora recomendações referentes à restrição do uso de telas na infância (33). Crianças menores de 2 anos não devem ter acesso a esses recursos; entre 2 e 5 anos, recomenda-se a permanência máxima de uma hora por dia e entre 6 e 10 anos, de até duas horas (33). Essa recomendação visa não somente diminuir a exposição da criança a propagandas, mas também evitar distrações no momento da refeição (33). Nessa mesma direção, a matriz sugere a realização de atividades lúdicas nas enfermarias pediátricas para reduzir o tempo de tela. Porém, para esta ação, o apoio dos profissionais e a disponibilização de um espaço adequado são pontos necessários e que podem representar barreiras para sua implementação. Nesse caso, a matriz pode ser utilizada como um instrumento de sensibilização em processos de negociação de mudança de rotinas e estruturais no ambiente hospitalar.

Outra ação que pode encontrar obstáculos para ser concretizada é a realização de orientações nutricionais voltadas à puérpera e à alimentação complementar da criança no banco de leite humano, pois esse local, cujo objetivo central é o apoio ao aleitamento materno e o manejo clínico da amamentação e nutrição do recém-nascido, não costuma dispor de profissionais para realizá-las.

Embora os nutricionistas sejam protagonistas das ações para promoção da alimentação adequada e saudável, o comprometimento de todos os profissionais de saúde é fundamental para que os Guias possam ser difundidos e implementados. As equipes interdisciplinares, ao compreenderem as recomendações dos Guias, podem incorporá-las ao seu trabalho, tornando-se suas multiplicadoras, estimulando a adoção de práticas alimentares adequadas e saudáveis pela população atendida e pelos trabalhadores que atuam no hospital. Assim, a formação dos profissionais para divulgarem as diretrizes alimentares é primordial para o sucesso de sua implementação (15). 

Ao considerar um hospital materno-infantil como base para a sua construção, a matriz remete à importância do olhar para este público, pois a qualidade da alimentação na gestação e nos primeiros anos de vida impacta no desenvolvimento infantil e na vida adulta, uma vez que o período intrauterino e os dois primeiros anos de vida são fases particularmente sensíveis a fatores metabólicos e nutricionais, que podem ocasionar consequências na saúde em curto e longo prazo (28). Nesse contexto, é necessário disseminar informações e propor ações voltadas à amamentação e alimentação adequada e saudável, o que reforça a importância da Matriz.

Ela apresenta barreiras e desafios a serem superados para que se torne uma realidade, isto é, para que as ações nela elencadas se materializem. A factibilidade da sua implementação não depende somente do engajamento e comprometimento dos trabalhadores, mas, principalmente, da equipe de gestão do hospital, que detém o poder de decisão para a implementação dessas ações.

Com a intencionalidade de traduzir as recomendações dos Guias para o cenário hospitalar, a matriz pode ser considerada ferramenta importante para orientar não somente a atuação dos profissionais de saúde na atenção nutricional no âmbito hospitalar do Sistema Único de Saúde ou do setor privado, como também para subsidiar o gestor do hospital para a criação de mecanismos que concretizem as orientações das diretrizes alimentares brasileiras. 

Algumas limitações devem ser consideradas na interpretação dos resultados. A primeira reside no tamanho da matriz. Como ela aborda trechos dos dois Guias, as suas duas seções são extensas. Contudo, a matriz tem a intenção de ser um produto detalhado para que cada serviço possa se apropriar da parte que considere necessária ou prioritária. Outra limitação é a ausência, na matriz, do setor de emergência e das máquinas de autosserviço. Cabe ressaltar que ela pode ser adaptada a cada realidade, podendo ser complementada com outros serviços e setores hospitalares relevantes em cada contexto.

A matriz apresenta interfaces necessárias para a concretização dos Guias Alimentares no contexto hospitalar materno-infantil, e em outros contextos semelhantes, evidenciando o seu potencial para a promoção da alimentação adequada e saudável, bem como para a qualificação do cuidado em alimentação e nutrição neste espaço.

## Data Availability

Não se aplica.
